# Prion Switching in Response to Environmental Stress

**DOI:** 10.1371/journal.pbio.0060294

**Published:** 2008-11-25

**Authors:** Jens Tyedmers, Maria Lucia Madariaga, Susan Lindquist

**Affiliations:** 1 Whitehead Institute for Biomedical Research, Cambridge, Massachusetts, United States of America; 2 Harvard-MIT Division of Health Sciences and Technology, Harvard Medical School, Boston, Massachusetts, United States of America; 3 Howard Hughes Medical Institute, Department of Biology, Massachusetts Institute of Technology, Cambridge, Massachusetts, United States of America; Howard Hughes Medical Institute, University of California San Francisco, United States of America

## Abstract

Evolution depends on the manner in which genetic variation is translated into new phenotypes. There has been much debate about whether organisms might have specific mechanisms for “evolvability,” which would generate heritable phenotypic variation with adaptive value and could act to enhance the rate of evolution. Capacitor systems, which allow the accumulation of cryptic genetic variation and release it under stressful conditions, might provide such a mechanism. In yeast, the prion [*PSI^+^*] exposes a large array of previously hidden genetic variation, and the phenotypes it thereby produces are advantageous roughly 25% of the time. The notion that [*PSI^+^*] is a mechanism for evolvability would be strengthened if the frequency of its appearance increased with stress. That is, a system that mediates even the haphazard appearance of new phenotypes, which have a reasonable chance of adaptive value would be beneficial if it were deployed at times when the organism is not well adapted to its environment. In an unbiased, high-throughput, genome-wide screen for factors that modify the frequency of [*PSI^+^*] induction, signal transducers and stress response genes were particularly prominent. Furthermore, prion induction increased by as much as 60-fold when cells were exposed to various stressful conditions, such as oxidative stress (H_2_O_2_) or high salt concentrations. The severity of stress and the frequency of [*PSI^+^*] induction were highly correlated. These findings support the hypothesis that [*PSI^+^*] is a mechanism to increase survival in fluctuating environments and might function as a capacitor to promote evolvability.

## Author Summary

One controversy in evolutionary biology concerns whether there might be plausible explanations for the rapid evolution of complex traits. An extreme and fascinating example of protein conformational change, the prion, offers a framework for this concept. Prion proteins are responsible for neurodegenerative diseases, instruct us in important aspects of amyloid formation, and furthermore, serve as ancient protein-based units of inheritance, a domain previously reserved for nucleic acids. In yeast, the [*PSI^+^*] prion causes read-through of nonsense codons. This has the capacity to rapidly unveil hidden genetic variation that may have adaptive value. The suggestion that [*PSI^+^*] might serve as a mechanism for evolvability would be strengthened if the frequency of the prion's appearance increased when the organism was under stress and therefore not ideally adapted to its environment. We investigated genetic and environmental factors that could modify the frequency with which the prion appears. Our high-throughput, genome-wide screen identified genes involved in stress response and signal transduction, whereas our cell-based assays found severe conditions that increased prion formation. Thus [*PSI^+^*] provides a possible mechanism for the organism to rapidly acquire new phenotypes in times of stress and potentially increases evolvability.

## Abstract 3

A yeast prion protein may promote evolvability by providing a mechanism for the rapid evolution of complex traits that is responsive to environmental stress.

## Introduction

[*PSI* ^+^] is an epigenetic modifier of translation termination in yeast [1,2]. It is formed by the protein Sup35, a subunit of the translation termination complex, which carries an intrinsically disordered prion-determining region at its N terminus. When this domain switches to an aggregating amyloid conformation (the prion conformation), much of the protein becomes unavailable for translation termination. Ribosomes therefore begin to read through stop codons an appreciable fraction of the time [3–6]. This creates a host of new phenotypes [7,8], because ribosomal read-through can cause changes in mRNA stability and protein function [9]. [*PSI* ^+^] generates different phenotypes in different genetic backgrounds as a result of the high levels of sequence variation downstream of stop codons. These phenotypes are heritable because the prion protein is passed through the cytoplasm to progeny, where self-templating conformational change perpetuates it [4,5,7,8]. Remarkably, under growth conditions in which [*PSI* ^+^] produces new phenotypes, these phenotypes are advantageous as much as ∼25% of the time [7].

Reducing the fidelity of translation termination should generally be deleterious. Not surprisingly, extensive sampling of Saccharomyces cerevisiae from the wild failed to recover any that were in the [*PSI* ^+^] state [10,11]. However, the unusual amino-acid composition of the prion domain of Sup35 (PD) has been conserved for over 800 million years of fungal evolution [4,12,13]. Although the sequence itself is poorly conserved, its ability to switch into a prion conformation and perpetuate that conformation in a stable heritable way [11,12,14], as well as the precise mechanism of conformational conversion [15,16] and the regulation of its conformations by the protein remodeling factor Hsp104 [17,18], have been conserved over the same period.

We have proposed that the transient appearance of [*PSI* ^+^] provides a mechanism for cells to acquire complex traits in a single step by sampling hidden genetic variation on a genome-wide scale. These complex traits can also be lost in a single step if the environments that favored them disappear, by simple loss of the prion conformation. Alternatively, when the selective pressure is maintained, [*PSI* ^+^] cells could propagate, allowing time for the traits to be fixed and further enhanced by genetic change [7,19]. In addition to increasing survival in fluctuating environments on a short time scale, this remarkable feature of the prion provides a framework for a mechanism for evolvability, which is the capacity of an organism to generate heritable phenotypic variation that has adaptive value [20].

However, the question of whether mechanisms for evolvability may themselves be the result of natural selection remains a hotly contested issue [21–23]. In considering whether [*PSI* ^+^] may be maintained over such long evolutionary distances because it was occasionally selected for by the evolutionary novelties it produced, it has been noted that there should be a correlation between the appearance of the prion and exposure to the stressful environments in which it is potentially advantageous [24–26].

Under typical laboratory conditions, [*PSI* ^+^] appears and is lost apparently spontaneously at low frequency (10^−6^–10^−7^ cells) [3,27,28]. Deliberate experimental manipulation of protein chaperones and degradation systems [29–32] can alter the frequency with which [*PSI* ^+^] appears. However, little is known about the relationship between de novo formation of [*PSI* ^+^] and stressful environments, except that prolonged storage at 4 °C increases the number of [*PSI* ^+^] cells [31,33]. Here we show that an increased frequency of [*PSI* ^+^] formation is linked to the cellular stress response, both by conducting an unbiased genome-wide screen for genetic modifiers of prion induction and by testing a large array of stressful growth conditions. We propose that the appearance and loss of [*PSI* ^+^] is intrinsically linked to perturbations in homeostatic mechanisms, particularly protein folding, simply because prion gain and loss are caused by rearrangements of an aggregation-prone, natively unfolded protein domain and is regulated by the activities of several different protein chaperones [5,32,34,35].

## Results

### A Genome-Wide Screen for Modifiers of [*PSI* ^+^] Induction

**Figure 1:**
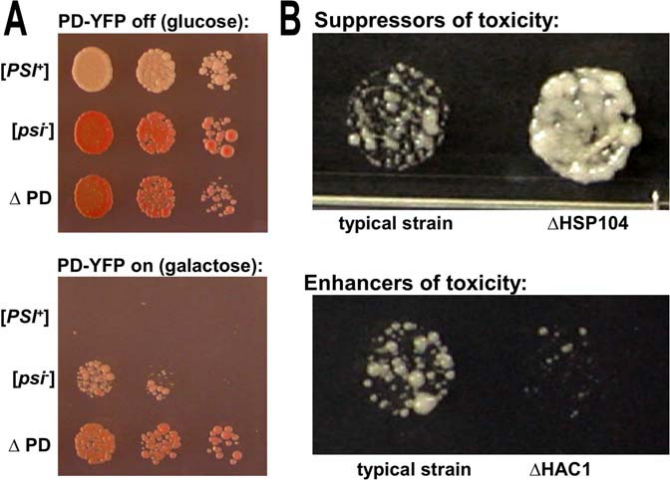
A Genome-Wide Screen for Modifiers of [*PSI* ^+^] Induction (A) The intermediate toxicity caused by overexpressing the prion domain in [*psi* ^–^] [*RNQ* ^+^] provides an opportunity to screen for modifiers of [*PSI* ^+^] induction. The prion domain (amino acids 1–253) of *SUP35* was fused to YFP with an 8–amino acids (GSRVPVEK) linker (PD-YFP). Three different 74D-694 [*RNQ* ^+^] derivatives express PD-YFP from genomic copies under control of the galactose-inducible promoter. The induction of PD-YFP on galactose medium is toxic in [*PSI* ^+^] cells. In [*psi* ^–^] cells partial toxicity is observed because only a fraction converts to [*PSI* ^+^]. Confirming that [*PSI* ^+^] induction is the cause of toxicity, cells carrying a *SUP35* gene with its prion domain deleted (ΔPD) are immune. Serial dilutions were plated on SD (glucose) or SGal (galactose) medium and incubated for 3 d at 30°C. (B) Partial toxicity allowed us to identify both enhancers and suppressors of [*PSI* ^+^] induction. We transformed 4,700 strains of the yeast gene deletion set (YGDS, Euroscarf) with a galactose-inducible PD-YFP 2μ plasmid in a high-throughput manner [67]. After selection for the plasmid, cells were spotted onto synthetic galactose-based medium to induce expression of PD-YFP. Some gene deletions cause a clear reduction of toxicity (decreased [*PSI* ^+^] induction, exemplified here by *hsp104*Δ), whereas others further enhance toxicity (increased [*PSI* ^+^] induction, exemplified by *hac1*Δ).

To conduct an unbiased genome-wide search for factors that influence prion formation, we took advantage of two long-standing observations: First, overexpressing the prion domain increases the frequency of [*PSI* ^+^] induction [36–38]. This is because the process of prion nucleation involves protein–protein interactions [35,39,40]. Second, continued overexpression, after cells have switched to the [*PSI* ^+^] state, is toxic. This is because overexpression drives too much of the essential Sup35 protein into the inactive prion state [36,38,41]. When the prion domain is overexpressed in [*psi* ^–^] cells, they will die upon switching to the [*PSI* ^+^] state (Figure 1A). This creates a toxicity that is proportional to prion induction frequency and therefore provides a means of screening for modifiers of induction frequency.

**Figure 2:**
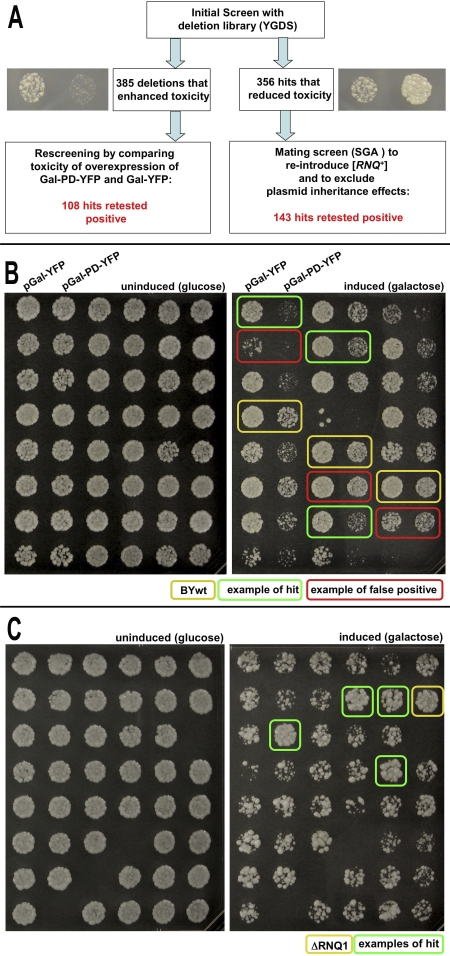
Large-Scale Secondary Screens to Retest Candidates (A) Schematic overview and summary of large-scale testing approach to verify screen hits. (B) Construct specificity in candidates with increased toxicity. Candidate deletion strains that showed increased toxicity in the screen were re-arrayed in duplicate columns in 96-well plates. One column was transformed with the construct coding for galactose-inducible PD-YFP and the second column was transformed with a plasmid coding for galactose-inducible YFP. To induce overexpression of YFP or PD-YFP, cells were pinned onto galactose (inducing) medium and onto glucose (non-inducing) medium as a control. Effects in deletion strains were then compared to the parental BY4741 strain (BYwt) of the deletion library (YGDS). (C) Strategy to retest suppressors of toxicity. We used the strategy of SGA [45] to combine two copies of the galactose-inducible PD-YFP with our candidate deletions that suppressed toxicity. The donor strain ([*RNQ* ^+^]) was mated to the candidate gene deletion strains by pinning onto plates containing the appropriate complete synthetic medium (SD). After overnight incubation, we replica-plated them four times onto medium selective for diploids. Diploids were then sporulated in liquid medium in 96-well plates at 23 °C for 6 d. Sporulation cultures were pinned and replica-plated three times onto selection plates for spores that combine the desired features. Overexpression of PD-YFP was achieved by spotting onto galactose medium, or glucose-based medium as a control.

We transformed each of the ∼4,700 strains in the S. cerevisiae single gene deletion library [42] (YGDS) with a galactose-inducible, plasmid-based expression construct for a prion-domain–yellow fluorescent protein fusion (PD-YFP). When transformants were switched from glucose to galactose, overexpression of PD-YFP caused an intermediate level of toxicity in most of the strains (Figure 1B, left). This allowed us to identify strains that enhanced or reduced toxicity (Figures 1B and 2A). As expected, the deletion of *hsp104* or *rnq1*, which was previously known to abolish [*PSI* ^+^] induction [18,43,44], eliminated the toxicity of PD-YFP overexpression (Figures 1B and 2B).

To eliminate candidate deletion strains that might have enhanced toxicity independently of [*PSI* ^+^] induction, we transformed them with a plasmid coding for YFP alone under control of the Gal promoter and compared the level of growth inhibition upon shift to galactose medium with that in strains carrying the PD-YFP fusion (Figure 2B). This left 108 strains (Table S1) that exhibited increased toxicity with PD-YFP but not with YFP (Figure 2B).

Next we eliminated candidate deletion strains in which the toxicity was reduced independently of changes in the [*PSI* ^+^] induction frequency, for example, by reducing the copy number of the 2μ plasmid used to induce the prion or by random loss of the prion-induction factor [*RNQ* ^+^] [38,43,44]. To do so, we integrated two copies of the galactose-inducible PD-YFP construct into the genome of a [*RNQ* ^+^] donor strain. These were introduced into the deletion strains by mating, sporulation and selection for the deletion [45] (Figure 2C). After retesting each strain (Figure 2C), we confirmed that 143 gene deletions reduced the toxicity of PD-YFP overexpression (Table S1).

### Functional Categorization of Screen Hits

The unexpectedly large number of genes that modify toxicity of PD-YFP overexpression indicates that prion induction frequency is highly responsive, directly or indirectly, to diverse cellular perturbations. To classify the candidate gene deletions, we used the Gene Ontology (GO) Slim Mapper program available at the S. cerevisiae genome database (SGD; http://db.yeastgenome.org/cgi-bin/GO/goSlimMapper.pl) for functional clustering analysis of the hits from our two groups: strains that enhanced toxicity and those that reduced toxicity of overexpression of PD-YFP.

For the reduced toxicity group, the most prominent categories were “cytoskeleton organization and biogenesis” (8% of hits versus 3% of library) and “cell budding” (6% of hits versus 1% of library) (Figure S1A). Several genes in these categories were found to alter the initial formation of prion foci, suggesting that they are involved in the mechanisms of prion formation and inheritance (JT and SL, unpublished data). For the enhanced toxicity group the most prominent categories were “signal transduction” (11% of hits versus 4% of library), “response to stress” (17% of hits versus 8% of library) and “response to chemical stimulus” (18% of hits versus 7% of library) (Figure S1B). This suggested a relationship between environmental stress and prion induction.

### Effects of Gene Deletions on Spontaneous [*PSI* ^+^] Induction Frequency

Next, we directly tested the deletions that changed toxicity of PD-YFP overexpression, together with some other closely related gene deletions, for effects on the spontaneous induction of [*PSI* ^+^]; that is, without overexpression of PD-YFP. These deletions were recreated by a site-directed knockout strategy [46] in a different strain background (74D-694 [*psi* ^–^]) to eliminate strain-specific effects and effects of genetic changes that may have accumulated in the original deletion library since it was created several years ago [47]. Because the rate of spontaneous induction of [*PSI* ^+^] is so low (10^−6^–10^−7^), we used a variant strain, R2E2, carrying a small insertion in the prion domain of Sup35 that increases [*PSI* ^+^] induction by facilitating conformational conversion in a protein-autonomous manner [28].

**Figure 3:**
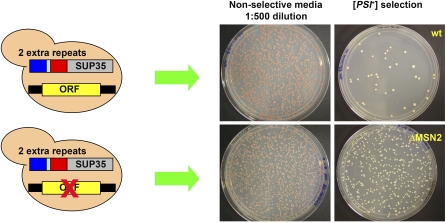
Measuring Spontaneous [*PSI* ^+^] Induction Frequency Candidate genes were knocked out in the repeat expansion strain 74D-694-R2E2 [28]. Cultures of different knockout strains and wild-type strains (150 μl for each) without any knockout were grown in 96-well plates for 48 h at 30 °C on a shaker. Each culture (12.5 μl) was plated directly onto adenine-deficient SD plates (SD-Ade), and a 1:500 dilution of the culture was plated onto complete SD plates as a control. Complete SD plates were incubated for 3 d at 30 °C and SD-Ade plates were incubated for 7 d at 30 °C. Colonies on the plates were counted using an Acolyte colony counter. Colonies on SD-Ade plates were tested for curability [50] by replica plating onto plates containing 3 mM guanidine HCl and subsequent reprobing on SD-Ade plates. Non-curable colonies were subtracted. For gene deletion strains, all ADE^+^ colonies were tested for curability, whereas for stress conditions, a representative subset was tested. Curability was always above 95%. Relative induction frequencies were calculated by dividing the number of colonies on SD-Ade plates by the number on complete synthetic medium.

Because of the labor involved, we focused on a subset of 40 genes from the best represented categories that appeared to have the most reproducible effects and did not have other confounding phenotypes (Table S2). Cells carrying a stop codon mutation in the *ADE1* gene were selected for the read-through phenotype characteristic of [*PSI* ^+^] by growth on medium deficient in adenine. An example of the data obtained is shown in Figure 3, which illustrates the effects of deleting the gene encoding the general stress-response transcription factor Msn2 [48,49]. Each genetic knockout was tested repeatedly to obtain induction rates with statistical significance. For each, we tested multiple independent transformants and all deletions were confirmed by colony PCR. Finally, we tested hundreds of colonies that appeared on the [*PSI* ^+^] selection plates for the presence of bona fide [*PSI* ^+^] elements by curing their read-through phenotypes with guanidine HCl [50].

**Figure 4:**
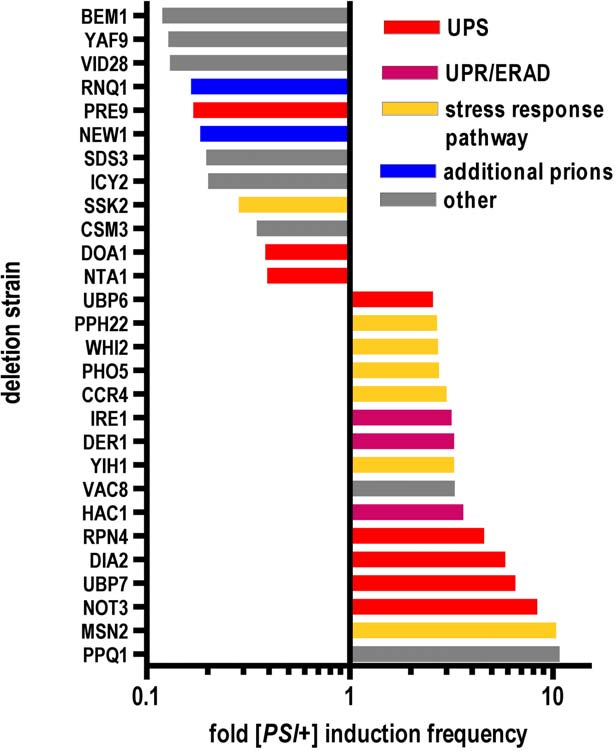
Gene Deletions That Affect [*PSI* ^+^] Induction Frequency Strains that were 74D-694-R2E2 [*psi* ^–^] [*RNQ* ^+^] [28] and that carried the 40 deletions that were tested most thoroughly were cured of any [*PSI* ^+^] elements that might have appeared spontaneously by growing cells carrying a 2μ plasmid carrying HSP104 regulated by a galactose-inducible promoter [18]. Cells were transferred to glucose medium in 96-well plates and grown for 48 h at 30°C. [*PSI* ^+^] induction frequency was determined first by plating aliquots onto selective medium (SD-Ade) followed by testing for curability by guanidine HCl, which is known to eliminate [*PSI* ^+^] [50] (compare Figure 3). The data shown are the results of seven independent experiments testing a total of at least six independent transformants for each deletion strain. The least square mean values of [*PSI* ^+^] induction frequency (*p* < 0.05 to *p* < 0.001) were plotted after normalizing their values to the wild type. The bars are coded to indicate different functional groups: UPS, ubiquitin-proteasome system; UPR, unfolded protein response; ERAD, endoplasmic-reticulum-associated degradation.

Of the 40 deletions tested in this manner, 16 deletions increased [*PSI* ^+^] induction frequency and 12 decreased it, both in a statistically significant manner (Student's *t-*test, *p* < 0.001–0.05, Figure 4). It was previously shown that members of the ubiquitin–proteasome system (UPS), such as Ubp6, Ubc4, and Doa4, influence [*PSI* ^+^] prion formation; for example, *ubc4*Δ facilitates spontaneous [*PSI* ^+^] formation [29,31]. Consistent with this, we detected additional members of the UPS that significantly altered [*PSI* ^+^] induction: the transcription factor Rpn4, which regulates expression of proteasome genes [51]; the ubiquitin-specific protease Ubp7; the only nonessential 20S subunit Pre9; and Doa1, which is involved in controlling cellular ubiquitin levels [52]. [*PSI* ^+^] induction frequency increased after deleting either the kinase Ire1 [53] or the transcription factor Hac1 [53], which are both regulators of the unfolded protein response (UPR), or Der1, a protein involved in endoplasmic-reticulum-associated degradation. Our findings also implicate proteins in other stress response pathways, such as phosphate starvation (Pho5), osmotic shock (Ssk2), and the general stress response (Msn2). Consistent with this, Whi2 regulates Msn2 and is required for the full activation of the general stress response [54]—deleting Whi2 also increased [*PSI* ^+^] induction frequency significantly (*p* < 0.05, Figure 4).

We note that genes that affect [*PSI* ^+^] induction frequency were originally identified in the deletion strain library, which carries a wild-type *SUP35* gene. However, to confirm the effects of some of the deletions on [*PSI* ^+^] induction frequency in a strain with neither Sup35 overexpression nor the R2E2 allele, we used a sensitive marker for [*PSI* ^+^]-mediated translational read-through: a fusion protein with a stop codon in front of a flow-cytometry-optimized green fluorescent protein (GFP) marker [55,56]. To validate this method, cells emerging from a wild-type [*psi* ^–^] culture with GFP fluorescence (suggestive of a switch to the [*PSI* ^+^] state) were sorted by fluorescence-activated cell sorting (FACS) and plated on nonselective medium, on medium that selects for [*PSI* ^+^] cells, and on medium that cures cells of [*PSI* ^+^] (Figure S2). Surprisingly, we observed a much higher frequency of [*PSI* ^+^] emergence by this method than expected from previous unsorted plating-based quantifications in our and other laboratories [3,27,28,57] and, indeed, than that obtained by direct plating of these cells. The higher rate of [*PSI* ^+^] appearance scored by the fluorescent method was confirmed as being due to the bona fide appearance of cells that could give rise to [*PSI* ^+^] colonies when plated after sorting (Figure S2). The apparent difference in induction frequency obtained by the two methods may be explained by an unstable, transient [*PSI* ^+^] transition state that we have recently observed during de novo [*PSI* ^+^] induction (JT, J. Dong, M. McCaffery, H. Saibil, B. Bevis, and SL, unpublished data). In any case, the effects of the deletion mutations on the induction frequency of [*PSI* ^+^] were confirmed by this method (Figure S3).

### Stressful Growth Conditions Increase [*PSI* ^+^] Induction Frequency

To directly test the relationship between prion emergence and environmental stress responses, we monitored the frequency of [*PSI* ^+^] formation in wild-type cells under conditions that cause stress: high salt concentrations, oxidative stress, endoplasmic reticulum stress, and high temperatures (Table S3). Identical aliquots of overnight cultures were diluted to 0.25 optical density units and incubated for various times under different test conditions (or in standard synthetic medium for control) and then quantified for [*PSI* ^+^] induction frequency (Figure 3). For each condition, three to five parallel samples were tested and each experiment was repeated several times. We tested both short intense stresses (shock) and longer extended stresses (12–24 h).

**Figure 5:**
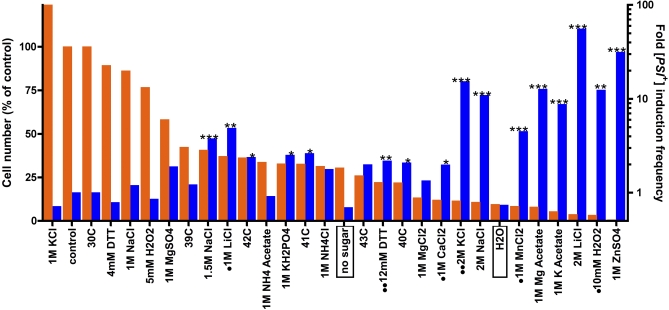
Stressful Growth Conditions Trigger Induction of the [*PSI* ^+^] Prion A culture of 74D-694-R2E2 was transferred to a 96-well plate at an optical density of 0.25 and incubated with complete synthetic medium as a control or medium supplemented with the indicated substances and incubated for 12–24 h at 30°C or higher temperatures as indicated. Cultures were subsequently plated on complete synthetic medium (SD) and on medium selective for [*PSI* ^+^] (SD-Ade) to determine the frequency of [*PSI* ^+^] induction (Figure 3). [*PSI* ^+^] status was confirmed by guanidine HCl curing. Orange bars, cell numbers determined on complete synthetic medium (left axis). Blue bars, [*PSI* ^+^] induction frequency (right axis). Conditions that significantly increased [*PSI* ^+^] induction relative to control are indicated by asterisks (*p < 0.05, **p < 0.01, ***p < 0.001). [*PSI* ^+^] cells had a growth disadvantage in conditions indicated by dots and had a growth advantage in conditions indicated by double dots. Significant increases in [*PSI* ^+^] induction frequency were determined as described in Materials and Methods.

Some stresses severely affected cell number, by killing cells or by inhibiting growth. Others did not. None of the short-term exposures showed a significant effect on the frequency of [*PSI* ^+^] induction (unpublished data). However, longer exposure to each of the different types of stress changed the [*PSI* ^+^] induction frequency significantly (*p* < 0.001–0.05). When conditions were sorted according to the severity of their effects on cell number (Figure 5, orange bars), a striking correlation (*p* < 0.0001) was observed between the severity of the stress and [*PSI* ^+^] induction frequency (Figure 5, blue bars). For example, 1 M NaCl did not influence growth or [*PSI* ^+^] induction. Increasing the concentration of NaCl to 1.5 M decreased cell number ∼2-fold and [*PSI* ^+^] induction frequency increased 4-fold. At 2 M NaCl, cell number decreased 10-fold and [*PSI* ^+^] induction frequency increased ∼10-fold. This trend was independent of the type of stress (Figure 5). Importantly, [*PSI* ^+^] induction was not just a consequence of reduced growth, but was associated with stress. Cultures maintained for 24 h in water or medium lacking sugar, which induce a quiescent state, also had low cell densities but had no increased induction of [*PSI* ^+^] (Figure 5, boxed).

If stress itself is the mechanism that increases switching to the [*PSI* ^+^] state, it should increase [*PSI* ^+^] induction irrespective of whether [*PSI* ^+^] would prove to be advantageous or disadvantageous in that condition. We determined the effect of each condition on the relative growth of [*PSI* ^+^] and [*psi* ^–^] cells. At the same time that initial cultures were prepared and tested for [*PSI* ^+^] induction frequency, we also pre-mixed a known ratio of wild-type [*PSI* ^+^] and [*psi* ^–^] cells, exposed them to the same test condition, and measured the ratio of the two cell types afterwards. As expected, in the majority of conditions tested, [*PSI* ^+^] was either neutral (Figure 5, unlabeled) or disadvantageous (Figure 5, single dots) for growth. In the latter case, in which [*PSI* ^+^] was at a selective disadvantage, the frequency of its induction was likely even higher. In two conditions, 12 mM DTT and 2 M KCl (Fig 5, double dots), [*PSI* ^+^] cells had a significant growth advantage over [*psi* ^–^] cells. In these cases [*PSI* ^+^] induction frequencies are likely somewhat inflated.

## Discussion

[*PSI* ^+^] formation is associated with the acquisition of new phenotypes by the exposure of previously hidden genetic variation [7]. Rather than forming stochastically with a steady low frequency, as previously supposed, we find that the frequency of [*PSI* ^+^] induction increases with stress. This was first suggested by the results of an unbiased screen of 4,700 viable gene deletions for changes in the frequency of [*PSI* ^+^] induction in a strain carrying a wild-type copy of the *SUP35* gene, in which [*PSI* ^+^] was induced by overexpression of the prion domain. The effects of the deletions were confirmed in a different strain background, which carried a mutation that allowed a direct quantitative measure of spontaneous induction. Finally, [*PSI* ^+^] induction frequencies were found to be increased by exposure to stressful environments, in a manner that correlated with the severity of the stress.

This stunning correlation fulfills a theoretical prediction: if [*PSI* ^+^] represents a mechanism for evolvability [7,19], its appearance should correlate with stress [23–26]. The inherent logic of the system derives from the fact that [*PSI* ^+^] is generally detrimental. First, it reduces the fidelity of protein synthesis. Given the many mechanisms that have evolved to ensure the fidelity of protein synthesis, this would be expected to have at least some detrimental effect on fitness, even if that effect is too small to be noticed in most laboratory situations. Second, in the majority of cases the hidden genetic variation that is revealed by [*PSI* ^+^] is either neutral or deleterious. Thus, under optimal growth conditions, the frequency of switching to the prion state is so low as to have a negligible effect on fitness (one in ∼10^6^–10^7^). Under conditions of extreme stress, when the expressed phenotype is poorly suited to the environment, an increase in the acquisition of [*PSI* ^+^] provides an opportunity for cells to alter the phenotypic read-out of their genome, providing an immediate access to complex traits [19] that have an appreciable chance of reaching a new optimum. However, in keeping with the fact that only a fraction of [*PSI* ^+^]-induced traits are beneficial, most cells in the culture still do not switch. Notably, in earlier studies, some of the same conditions that we found to increase the frequency of [*PSI* ^+^] (2 M KCl or high concentrations of MgAc_2_, as well as other types of stress) were reported to facilitate the loss of [*PSI* ^+^] [50,58]. The same logic applies here. If the expressed phenotype is well suited to the environment, [*PSI* ^+^] is relatively stable, but if it is not, the frequency of switching increases.

Mechanistically, how might this be accomplished? It was recently reported that challenging the capacity of protein quality control in *Caenorhabditis elegans,* by overexpressing folding-defective mutant proteins, enhances the aggregation of other glutamine-rich amyloid-forming proteins, such as a polyglutamine-expanded fragment of huntingtin [59]. By analogy, challenges to protein homeostasis by severe stress may stabilize partially folded or misfolded Sup35 molecules, as well as other glutamine-rich proteins [43,44,60], and facilitate nucleated conformational conversion to the prion state [35]. Furthermore, a complex web of interactions between Sup35 and the chaperone network of the cell controls the assembly and disassembly of the prion amyloid in vitro ([4,32,61],). The complex and varied nature of these interactions can plausibly explain increased switching to [*psi* ^–^] in cells that are [*PSI* ^+^] and vice versa. Consider, for example, the stress-induced heat shock protein Hsp104, a protein remodeling factor that conformationally remodels the [*PSI* ^+^] determinant Sup35 via several mechanistically distinct mechanisms. High concentrations of Hsp104, which occur with stress, promote disassembly of Sup35 prions, whereas ADP, whose concentration rises during stress, reduces the inhibition of prion nucleation by Hsp104 [4,18]. These interactions represent an intrinsic mechanism by which the state of protein homeostasis increases the likelihood that cells will switch from [*psi* ^–^] to [*PSI* ^+^], or from [*PSI* ^+^] to [*psi* ^–^], when the cell is not well adapted to its environment.

But how might the prion have appeared in the first place? The extreme enrichment of the prion domain in glutamine residues suggests that it may simply have arisen as a polyglutamine expansion, an event that occurs frequently in eukaryotic genomes [62]. Simple stretches of polyglutamine can provide new functions [63] but can be toxic in their own right if they expand too far [64], and this would have created selective pressure for the sequence to diverge away from simple polyglutamine while preserving other possible functions, as suggested by evidence of purifying selection operating on the prion-forming domain of *SUP35* [12]. Together, these observations provide a credible conceptual framework for the evolution of a system for enhancing evolvability. Other systems for evolvability may include mechanisms that increase sex and recombination, and thereby variation, in response to stress [65]. The ability of the molecular chaperone Hsp90 to buffer hidden genetic variation and release it in response to stressful environments is another example [22,23,66]. These, too, may not initially have appeared because of their effects on evolvability per se, but may simply have arisen as an inherent consequence of effects of stress on genome stability and protein homeostasis.

## Materials and Methods

### Yeast strains and media.

We used the BY4741 deletion set provided by EUROSCARF [42], 74D-694 [18], and a 74D-694-R2E2 variant [28]. The media were complete standard synthetic medium or medium lacking particular amino acids and containing either d-glucose (SD) or d-galactose (SGal) as sole carbon source. Sporulation was performed in liquid rich sporulation medium (1% potassium acetate, 0.05% dextrose, 0.1% yeast extract, and 0.01% complete amino acid mix, Bio101)

### Gene deletions of candidates in 74D-694-R2E2.

Primer sequences to create knockouts were from the yeast deletion project (http://www-sequence.stanford.edu/group/yeast_deletion_project/deletions3.html). Knockout cassettes were generated by PCR using the kanMX4 plasmid pFA6a [46] and individual knockout primers. Yeast were transformed with purified PCR products using lithium acetate. The knockouts were confirmed using the KanB primer [46], which primes in the kanMX4 cassette, together with individual 20–22mers ∼700 bp upstream of the start codons of the candidate genes.

### Direct measurement of spontaneous [*PSI* ^+^] induction frequency.

Cultures were grown as described in each figure. An aliquot was plated onto SD- plates lacking adenine (SD-Ade) and incubated for 7–9 d at 30 °C, whereas a 1:500 dilution of the culture was plated onto complete SD plates for total cell number determination and incubated for 3 d. Non-curable colonies were excluded before calculating [*PSI* ^+^] induction frequency (ratio of [*PSI* ^+^] colonies to total cell number) as described in Figure 3.

### Screen for modulators of [*PSI* ^+^] induction.

High-throughput transformations were done in a 96-well format [67]. Transformants were selected for 3 d at 30°C on SD plates lacking uracil (SD-Ura). Growth effects of PD-YFP overexpression were determined by replica plating on SGal-Ura and incubation for 3 d at 30 °C. Effects from the deletion itself were excluded by plating on SD-Ura in parallel.

### Retesting of candidates in secondary screens.

Candidate deletion strains were re-arrayed in two different sets in 96-well plates: increased and decreased toxicity of PD-YFP overexpression. To retest deletion strains with decreased toxicity (using systematic gene analysis, SGA [45]), we mated a haploid Mat-alpha [*RNQ* ^+^] donor strain containing two genomic copies of galactose inducible PD-YFP with the deletion library strains of interest in 96-well format. After pinning the two strains together on YPD plates and incubating them overnight at 30 °C, we spotted the cells four consecutive times on SD medium containing 200 μg/ml geneticin (for selection of kanMX cassette of the deletion strain) to select for diploids. Diploids were subjected to sporulation in liquid medium and incubated for 6 d at 23 °C. Haploid spores of interest were selected for by spotting onto selective SD medium three consecutive times [45] and incubation for 3 d at 30 °C each time. PD-YFP expression was induced by spotting onto selective SGal plates and on selective SD plates as control and incubated at 30 °C for 3–4 d.

To retest deletion strains with increased toxicity, cells were transformed with a 2μ plasmid coding for PD-YFP or just YFP under control of the galactose promoter. To induce expression, cells were pinned onto synthetic selective SGal medium and selective SD medium as a control and incubated at 30 °C for 3–4 d, respectively.

### Statistical methods.

 Calculated [*PSI* ^+^] induction frequencies were log transformed to improve normality. Multiple linear regression analyses were performed with experiment and genotype (Figure 4) or experiment, condition, and time (Figure 5) as fixed effects. Significance (α < 0.05) was determined by a two-tailed Student *t*-test. Exact *p*-values were calculated from the *t*-ratio of each effect. Effects were normalized to control (wild-type strain or untreated sample) to demonstrate fold increase or decrease.

## Supporting Information

Figure S1 Functional Categorization of Screen Hits (A and B) The candidate genes were assigned to different functional categories using the GO Slim Mapper program in the S. cerevisiae genome database (http://db.yeastgenome.org/cgi-bin/GO/goSlimMapper.pl). (A) Candidate gene deletions that decrease the toxicity of overexpression of PD-YFP. Clustering of candidate genes as compared with the abundance in the library was found in categories such as “cytokinesis,” “cell budding,” “cell cycle,” “meiosis,” or “conjugation” as well as in “cytoskeleton organization and biogenesis” or “vesicle mediated transport.” (B) Candidate gene deletions that increase the toxicity of overexpression of PD-YFP. Clustering of candidate genes was found most prominently in functional categories such as “transcription,” “signal transduction,” “response to stress,” “response to chemical stimulus,” or “protein modification.”
(4.5 MB JPG)

Figure S2 FACS of Wild-Type [*psi^–^*] Cells Containing a GFP Read-Through Marker [*psi^–^*] cells (74D-694) were transformed with a fusion protein containing a FACS-optimized GFP marker [56] preceded by a stop codon [55] (briefly, the ds-Red marker [55] was switched with the GFP marker [56]). Cells were grown for 24 h and sorted into GFP-positive and gfp-negative cells (top left panel). The right column shows GFP-positive colonies (suggestive of a switch to the [*PSI^+^*] state) that appear white on non-selective plates (YPD), grow on SD-Ade plates (selective for [*PSI^+^*]), and are curable by guanidine HCl. The left column of plates shows gfp-negative colonies ([*psi^–^*] cells) that appear red on YPD plates and do not grow on SD-Ade plates.
(1.5 MB JPG)

Figure S3 Gene Deletions That Affect [*PSI^+^*] Induction Frequency in Wild-Type [*psi^–^*] Cells Deletions were made in [*psi^–^*] cells (74D-694) containing a FACS-optimized GFP marker preceded by a stop codon integrated at the *URA* locus. Independent transformants were picked and grown in 150-μl SD-Ura in 96-well plates for 24 h. The cultures were stained with propidium iodide to exclude dead cells and debris before quantifying the number of GFP-positive and -negative cells by flow cytometry. Three independent experiments with triplicate independent transformants were analyzed by calculating the percentage of GFP-positive cells, and a Wilcoxon test was used to determine the gene deletions that significantly altered [*PSI* ^+^] induction (*p* < 0.05). The mean values for each significant deletion were then normalized to the wild-type control and plotted on a logarithmic scale.
(350 KB JPG)

Table S1 Gene Deletions from the YGDS That Reduced or Enhanced the Toxicity of PD-YFP Overexpression (107 KB DOC)

Table S2 Genes Knocked Out in 74D-694-R2E2 to Test Their Effect on Spontaneous Induction Frequency (33 KB DOC)

Table S3 Summary Table of Conditions Tested for Their Effect on the Induction Frequency of [*PSI* ^+^] (36 KB XLS)

## Abbreviations

FACS: fluorescence-activated cell sorting
PD-YFP: prion-domain yellow fluorescent protein fusion
UPS: ubiquitin-proteasome system
