# Supplementary figures and images for: Prion Switching in Response to Environmental Stress
Source: PLoS Biol. 2008 Nov 25;6(11):e294. doi: 10.1371/journal.pbio.0060294 (PMC2586387; doi:10.1371/journal.pbio.0060294)

## Reduced toxicity PD-YFP overexpression

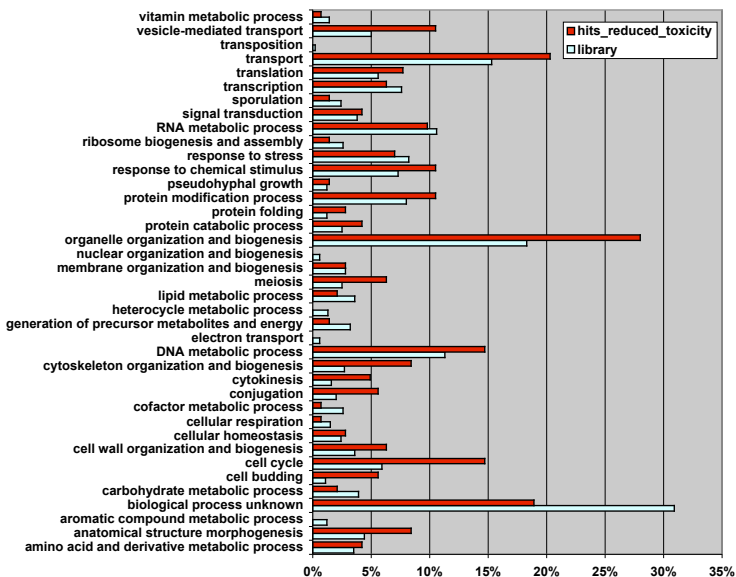

## Enhanced toxicity PD-YFP overexpression

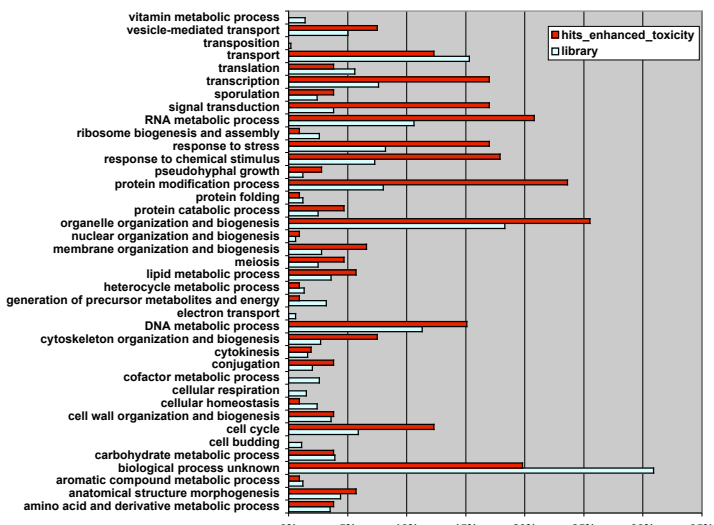

Supplement: Figure S1 — (A and B) The candidate genes were assigned to different functional categories using the GO Slim Mapper program in the S. cerevisiae genome database (http://db.yeastgenome.org/cgi-bin/GO/goSlimMapper.pl). (A) Candidate gene deletions that decrease the toxicity of overexpression of PD-YFP. Clustering of candidate genes as compared with the abundance in the library was found in categories such as “cytokinesis,” “cell budding,” “cell cycle,” “meiosis,” or “conjugation” as well as in “cytoskeleton organization and biogenesis” or “vesicle mediated transport.” (B) Candidate gene deletions that increase the toxicity of overexpression of PD-YFP. Clustering of candidate genes was found most prominently in functional categories such as “transcription,” “signal transduction,” “response to stress,” “response to chemical stimulus,” or “protein modification.” (4.5 MB JPG) [file pbio.0060294.sg001.pdf]

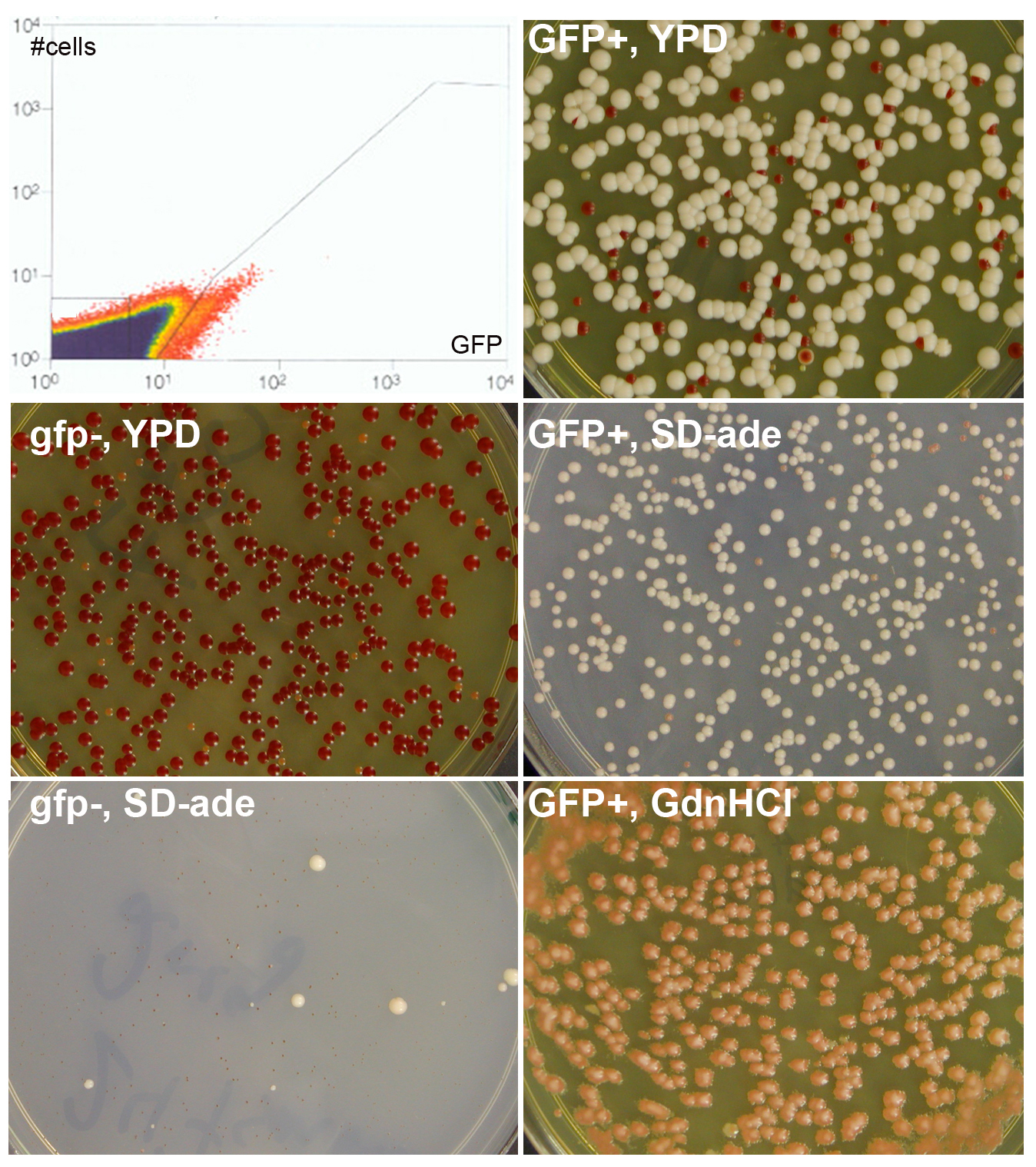

Supplement: Figure S2 — [psi–] cells (74D-694) were transformed with a fusion protein containing a FACS-optimized GFP marker [56] preceded by a stop codon [55] (briefly, the ds-Red marker [55] was switched with the GFP marker [56]). Cells were grown for 24 h and sorted into GFP-positive and gfp-negative cells (top left panel). The right column shows GFP-positive colonies (suggestive of a switch to the [PSI+] state) that appear white on non-selective plates (YPD), grow on SD-Ade plates (selective for [PSI+]), and are curable by guanidine HCl. The left column of plates shows gfp-negative colonies ([psi–] cells) that appear red on YPD plates and do not grow on SD-Ade plates. (1.5 MB JPG) [file pbio.0060294.sg002.jpg]

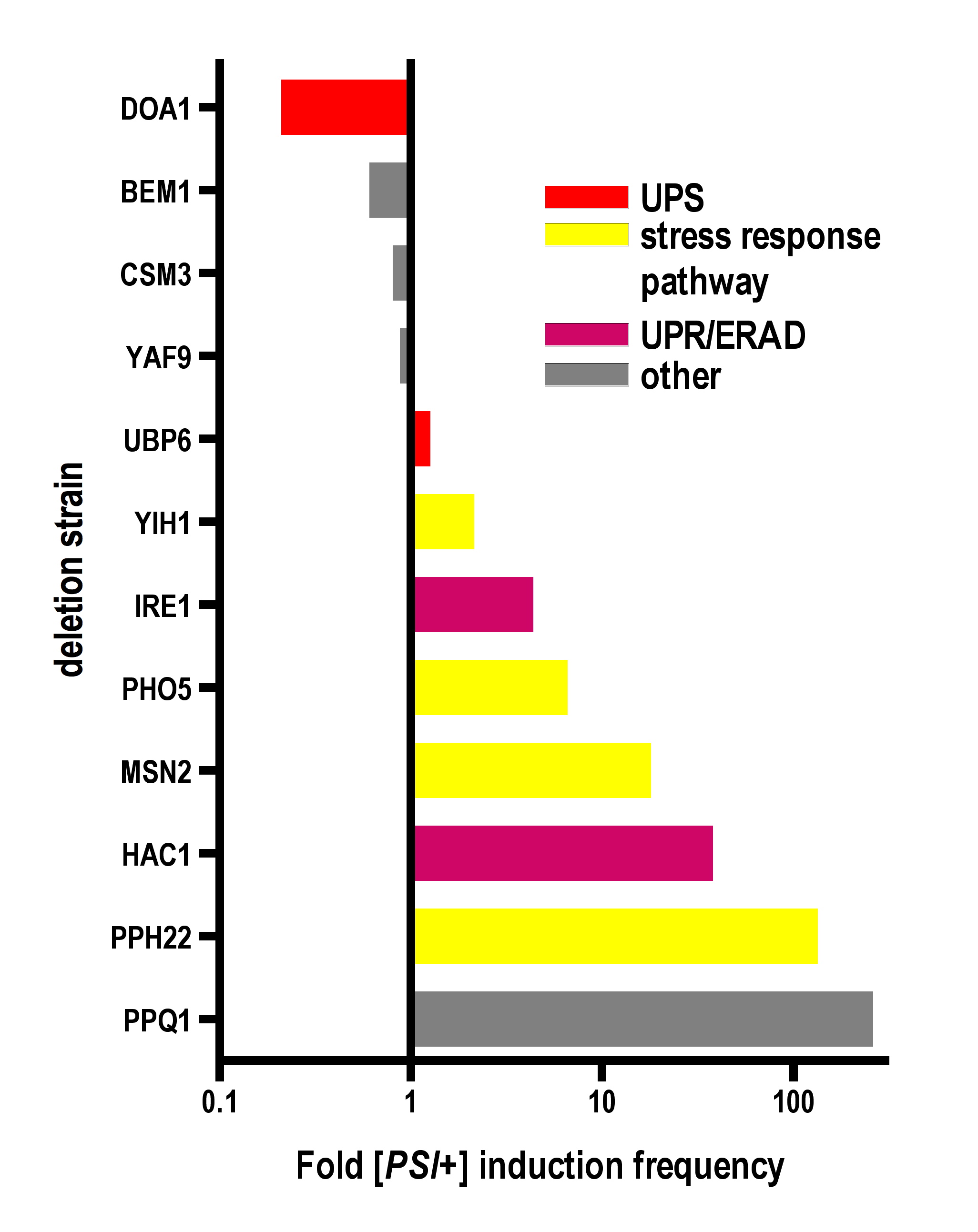

Supplement: Figure S3 — Deletions were made in [psi–] cells (74D-694) containing a FACS-optimized GFP marker preceded by a stop codon integrated at the URA locus. Independent transformants were picked and grown in 150-μl SD-Ura in 96-well plates for 24 h. The cultures were stained with propidium iodide to exclude dead cells and debris before quantifying the number of GFP-positive and -negative cells by flow cytometry. Three independent experiments with triplicate independent transformants were analyzed by calculating the percentage of GFP-positive cells, and a Wilcoxon test was used to determine the gene deletions that significantly altered [PSI +] induction (p < 0.05). The mean values for each significant deletion were then normalized to the wild-type control and plotted on a logarithmic scale. (350 KB JPG) [file pbio.0060294.sg003.jpg]
