# Supplementary material for: Prion Switching in Response to Environmental Stress
Source: PLoS Biol. 2008 Nov 25;6(11):e294. doi: 10.1371/journal.pbio.0060294 (PMC2586387; doi:10.1371/journal.pbio.0060294)
Supplement: Table S1 — (107 KB DOC) [file pbio.0060294.st001.doc]

Table S1: Genes deletions from the YGDS that reduced or enhanced the toxicity of PD-YFP overexpression.

| **Deletions enhancing toxicity (108)** | | | |
| --- | --- | --- | --- |
| ACF4 | HIR3 | RDS2 | UME6 |
| ADA2 | HOG1 | RHR2 | UNG1 |
| ALF1 | IRE1 | RPL2A | VAC8 |
| ARC18 | IZH4 | RPL41B | VID28 |
| ARE1 | KRE1 | RPN10 | VPS21 |
| ARN2 | LRP1 | RPS9B | WHI2 |
| BCK1 | MAL11 | RRI2 | WSC3 |
| BRE1 | MDM20 | RUP2 | YDL032W |
| CAF4 | MED1 | SAC1 | YDL183C |
| CCR4 | MET6 | SDC1 | YDR537C |
| CDC26 | MIP6 | SGF73 | YER128W |
| CST9 | MLC2 | SGN1 | YFL032W |
| CWH41 | MNI1 | SIC1 | YGR050C |
| DAP2 | MNT4 | SKP2 | YGR250C |
| DEG1 | MRE11 | SLG1 | YHR087W |
| EAF7 | MSN2 | SPO22 | YIL028W |
| EDE1 | NAS2 | SRL3 | YIL092W |
| EPT1 | NOT3 | SSK2 | YIL168W |
| FKH2 | NPR1 | SST2 | YJR088C |
| FYV1 | NUP60 | STE20 | YJR129C |
| FYV12 | NUP84 | STE50 | YLL007C |
| GOS1 | OTU2 | SWA2 | YMR1 |
| GPA2 | PDE2 | SWD3 | YNL095C |
| GVP36 | PEX6 | SWE1 | YOX1 |
| GYP1 | PKP1 | TPK1 | YPR096C |
| HAC1 | PMT2 | TRP3 | YPR170C |
| HAP2 | PPQ1 | UBP8 | YPR197C |

Table S1 continued:

| **Deletions reducing Toxicity (143)** | | | |
| --- | --- | --- | --- |
| ADY2 | FAR3 | NTA1 | SPR28 |
| AIM3 | FAR7 | OSM1 | SPT4 |
| AIM44 | FIG1 | PCI8 | SUE1 |
| AKL1 | FLC2 | PIF1 | SUR4 |
| APC9 | FUS1 | PML39 | SWC3 |
| APL5 | GET3 | PNS1 | TED1 |
| APM3 | GIC2 | PPG1 | THI7 |
| APS2 | GIS1 | PPH21 | THI72 |
| APS3 | GPM2 | PPH22 | TIF4632 |
| ARF1 | GRX3 | PSK2 | TOS2 |
| ARF3 | HHT1 | PTK2 | TYE7 |
| ARG81 | HIS4 | PUB1 | UBP1 |
| ARP6 | HLJ1 | PUF3 | UBS1 |
| ARX1 | HOP2 | RAX1 | VTC3 |
| ASE1 | HPA3 | RDH54 | YAF9 |
| ASH1 | HRK1 | RGA1 | YBR012C |
| BAP2 | HSP104 | RNQ1 | YBR016W |
| BBC1 | IDS2 | RPL19B | YCR101C |
| BEM1 | INP51 | RPL1a | YCT1 |
| BFR1 | IRC10 | RPL22B | YDR307W |
| BOI2 | IRC2 | RPS23b | YGR018C |
| BZZ1 | KIP1 | RPS7b | YGR139W |
| CAF20 | KRE11 | RRF | YIP4 |
| CSM3 | LDB16 | RSR1 | YJR115W |
| CUE5 | MBF1 | RTS1 | YKL047W |
| CUP9 | MET3 | SAS4 | YKR015C |
| DCW1 | MET10 | SCJ1 | YKU80 |
| DEF1 | MET16 | SDS3 | YLL020C |
| DLT1 | MPC54 | SER33 | YLR050C |
| DSE1 | MSB3 | SLM2 | YLR437C |
| EGT2 | MSL1 | SNA2 | YML033W |
| EMC21 | MUD1 | SNA3 | YMR086W |
| EMC40 | NAT4 | SNX4 | YNL035C |
| EST2 | NKP1 | SPC1 | YNL198C |
| FAR10 | NOP13 | SPO1 | ZIP1 |
| FAR11 | NPY1 | SPO13 |  |
