# Supplementary material for: Prion Switching in Response to Environmental Stress
Source: PLoS Biol. 2008 Nov 25;6(11):e294. doi: 10.1371/journal.pbio.0060294 (PMC2586387; doi:10.1371/journal.pbio.0060294)
Supplement: Table S2 — (33 KB DOC) [file pbio.0060294.st002.doc]

Table S2: Genes knocked out in 74D-694 R2E2 to test their effect on spontaneous induction frequency

| ATG11 | HRK1 | PPH22 | SSK2 |
| --- | --- | --- | --- |
| BEM1 | ICY2 | PPQ1 | UBP6 |
| CCR4 | IRE1 | PRE9 | UBP7 |
| CSM3 | LDB16 | RNQ1 | VAC8 |
| DER1 | MSN2 | RPL19 | VID28 |
| DIA2 | NEW1 | RPN4 | VID30 |
| DOA1 | NOT3 | SDS3 | VPS9 |
| GID8 | NTA1 | SEC66 | WHI2 |
| HAC1 | PHO5 | SGF11 | YAF9 |
| HOG1 | PPG1 | SSD1 | YIH1 |
